# Genetic risk scores of psychiatric phenotypes are associated with depression risk in a prospective Dutch population-based cohort

**DOI:** 10.1017/S0033291725101943

**Published:** 2025-09-29

**Authors:** Amy Hofman, Bahar Sedaghati-Khayat, Annabel Vreeker, M. Arfan Ikram, André G. Uitterlinden, Joyce B.J. van Meurs, Jeroen G.J. van Rooij, Annemarie I. Luik

**Affiliations:** 1 Department of Epidemiology, Erasmus MC University Medical Center Rotterdam, Rotterdam, The Netherlands; 2 Department of Internal Medicine, Erasmus MC University Medical Center Rotterdam, Rotterdam, The Netherlands; 3 Department of Child and Adolescent Psychiatry/Psychology, Erasmus MC University Medical Center Rotterdam, Rotterdam, The Netherlands; 4 Department of Psychology, Education and Child Studies, Erasmus University Rotterdam, Rotterdam, the Netherlands; 5 Department of Orthopaedics and Sports Medicine, Erasmus MC University Medical Center Rotterdam, Rotterdam, The Netherlands

**Keywords:** clinical management, depression, genetic risk score, population-based, prevention

## Abstract

**Background:**

Genetic risk scores hold potential for predicting depression in the general population. These scores must be validated for their associations with relevant characteristics of depression-related phenotypes, such as severity. We validated a genome-wide risk score (GRS) and a restricted polygenic risk score (PRS) for depression based on a meta-analysis of three genome-wide association studies and assessed their associations with depression in three subcohorts of middle-aged and older adults from the Dutch population-based Rotterdam Study.

**Methods:**

Of participants with genotype data, 9,198 had longitudinally measured data (mean follow-up: 11.3 years) on three depression-related phenotypes (depressive symptoms, depressive syndrome, and major depressive disorder). Generalized linear models estimated the associations of standardized GRS and PRS with depression phenotypes per subcohort and were then meta-analyzed. One unit of the GRS/PRS represents 1 standard deviation, following *z*-transformation per cohort.

**Results:**

A one unit higher GRS and PRS were associated with any longitudinally measured depression phenotype (odds ratio (OR)_GRS_ = 1.20 [1.15–1.26], OR_PRS_ = 1.10 [1.05–1.16]). Effect sizes were highest for episodes of major depressive disorder: for individuals with the 10% highest GRS and PRS, the ORs were 1.99 [1.53–2.57] and 1.51 [1.13–1.99], respectively, compared to the middle 50% of the distribution.

**Conclusions:**

The GRS and PRS for depression showed modest associations across multiple depression-related phenotypes in a population-based setting. The strength of associations generally increased with the severity of the phenotype. While effect sizes were generally larger for GRS compared to PRS, the difference was mostly not statistically significant.

## Introduction

Depressive disorders represent the highest disease burden globally in terms of years lived with disability (World Health Organization, 2017). Their heterogeneous symptom profiles complicate diagnosis and the development of prevention and intervention strategies (Zimmerman et al., 2015). Also, subclinical symptoms are highly common, creating a burden on individuals and society, and these symptoms might develop into clinical diagnoses (Murray et al., 2021; Shah et al., 2020). Successfully addressing the phenotypic diversity of depression demands a thorough comprehension of its multifaceted presentations and associated genetic and environmental risk factors.

Family-based studies have consistently shown that major depressive disorder has a heritability estimate ranging from 30 to 40% (Polderman et al., 2015; Sullivan et al., 2000). Genome-wide association studies (GWAS) underscore the highly polygenic nature of the disorder, characterized by the involvement of numerous common variants, each contributing small increments of risk, jointly explaining 5–6% of variance in European cohorts (Als et al., 2023; Howard et al., 2019; Levey et al., 2021; McIntosh & Lewis, 2024; Meng et al., 2024; Wray et al., 2018). Subsequently, polygenic risk scores have been developed to quantify individual genetic liability (Als et al., 2023; Halldorsdottir et al., 2019; Howard et al., 2019; Kosciuszko et al., 2023; Kwong et al., 2021a; McIntosh & Lewis, 2024; Mitchell et al., 2021; Musliner et al., 2019; Wray et al., 2018). However, their predictive power remains limited, and their clinical utility needs further study.

Although these genetic risk scores have been studied in clinical and, to lesser extent, in population-based samples, the majority of studies focused on cohorts with younger participants (up to 35 years of age, including children) (Halldorsdottir et al., 2019; Kwong et al., 2021a; Musliner et al., 2019), or on broad age ranges across adulthood (Als et al., 2023; Howard et al., 2019; McIntosh & Lewis, 2024; Mitchell et al., 2021; Wray et al., 2018). Studies into genetic risk assessment in middle-aged and older adults remain limited, despite the ageing population globally and a peak of depression prevalence in middle and late adulthood (World Health Organization, 2017). Although twin studies generally suggest stable heritability estimates across adulthood, the relative influence of genetic factors may decrease as environmental factors play an increasingly important role at older ages (Kendler et al., 2011; Nivard et al., 2015). Consistently, polygenic risk scores have shown stronger associations with early-onset than late-onset depression (Power et al., 2017). Moreover, depression in late life may involve different biological mechanisms and symptom profiles, including somatic and neurodegenerative processes (Schaakxs et al., 2017; Szymkowicz et al., 2023), further emphasizing potential age-related variation in genetic liability.

In this context, Kosciuszko et al. (2023) showed that genetic risk scores are related to depressive symptoms in adults aged 50 and over, but only used one self-reported depression phenotype based on the Center for Epidemiological Studies Depression (CES-D) scale (Kosciuszko et al., 2023). However, previous studies have shown that associations may differ depending on depression phenotype, episode severity, or the presence of situational triggers, such as the COVID-19 pandemic (Halldorsdottir et al., 2019; Huang et al., 2023; Kwong et al., 2021a; Musliner et al., 2021; Sullivan et al., 2000; Wray et al., 2018). Therefore, an extensive assessment of genetic risk scores across phenotypes and characteristics of depression in middle and older age is warranted.

In this study, we aimed to evaluate the predictive capabilities of genetic risk scores based on the summary statistics of the study by Howard et al. (2019). We constructed and validated two commonly used types of genetic risk scores: a clumping and thresholding (C + T) genome-wide risk score (GRS) and a restricted polygenic risk score (PRS), including variants meeting the genome-wide significance (p < 5 × 10^–8^) for depression. While the GRS aggregates a broad range of genetic variants across the genome and may offer greater predictive power, the PRS is more interpretable and potentially easier to translate to clinical or biological contexts. This dual approach allows us to explore whether the GRS and PRS are differentially associated with the occurrence of depression-related phenotypes in a prospective cohort of middle-aged and older Dutch adults. Additionally, we examined whether associations differed depending on when and how depression-related phenotypes were measured: standard cross-sectional symptom reports, cross-sectional symptoms during the COVID-19 pandemic as an acute environmental stressor, and longitudinal data (follow-up >10 years) using clinically defined phenotypes.

## Methods

### Study design and population

This study was embedded within the population-based Rotterdam Study, a prospective cohort of adults aged 40 years and over that originated from 1989 (Ikram et al., 2024). The Rotterdam Study (RS) consists of four subcohorts of which three were included in this study: RS-I (started from 1989, *N* = 7,983 aged 55 years and over at initial recruitment), RS-II (started from 2000, N = 3,011 aged 55 years and over), and RS-III (started from 2006, N = 3,932 aged 45 years and over). Follow-up visits occur every 5 years on average.

In total, 9,282 participants from the three subcohorts were included in this study, with data available on genotype and any of the depression-related phenotypes of interest. Of these, a total of 7,316 participants contributed cross-sectional measured data on depression between 2002 and 2008. Longitudinal data were available for 9,198 participants based on continuous monitoring of medical records and additional assessments during study waves: RS-I (1993–2012, four waves), RS-II (2000–2012, three waves), and RS-III (2006–2019, two waves). Additionally, during a period in the COVID-19 pandemic (April 2020–July 2020), we collected information on depressive symptoms in 2,955 participants with genotype data available (Licher et al., 2021).

The Rotterdam Study has been approved by the Medical Ethics Committee of Erasmus MC (registration number MEC 02.1015) and by the Dutch Ministry of Health, Welfare and Sport (Population Screening Act WBO, license number 1071272–159521-PG). The Rotterdam Study Personal Registration Data collection is filed with the Erasmus MC Data Protection Officer under registration number EMC1712001. The Rotterdam Study has been registered at the Netherlands National Trial Register (NTR; www.trialregister.nl) and the WHO International Clinical Trials Registry Platform (ICTRP; www.who.int/ictrp/network/primary/en/) under shared catalog number NTR6831. All participants provided written informed consent to participate in the study and to have their information obtained from treating physicians.

### Measurements

#### Genotyping and genetic scores calculation

Genotyping of data within the Rotterdam Study cohorts has been described previously (Ikram et al., 2020). In short, samples were genotyped using Illumina’s 550 k or 610 k platform and imputed to the 1KGp3v5 reference panel.

The restricted PRS contains 102 independent SNPs with genome-wide significance (p < 5 × 10^−8^) selected from a large GWAS on depression, which included populations with a wide age range across adulthood (Howard et al., 2019). We used the same study summary statistics to generate the GRS. Linkage disequilibrium clumping (r2 < 0.1 in a 1-Mb window) was performed on any overlapping SNPs with the 1000 Genomes Project European samples for reference. GRS was calculated using PLINK (v1.9) for eight different scores based on different p-value thresholds (p ≤ 5 × 10^−8^, p ≤ 1 × 10^−5^, p ≤ 1 × 10^−3^, p ≤ 0.01, p ≤ 0.05, p ≤ 0.1, p ≤ 0.5, and p ≤ 1). The threshold yielding the highest variance explained (R^2^) across three RS subcohorts was selected for the calculation of the genome-wide risk score.

The average weighted score of each GRS and PRS is determined for each participant based on the following formula:

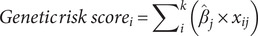

where genetic risk score_i_ refers to the genetic score for subject i, 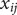 is the posterior probability of the alternate allele by a subject i of a variant j based on best-guess imputations, κ is the number of independent variants in the genetic risk score for subject i, and 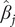 is the weight for variant j obtained from GWAS summary statistics. Both GRS and PRS were standardized to a mean of zero and a standard deviation of one (z-transformation) for each subcohort.

#### Depression

##### Cross-sectional data

Depressive symptoms were assessed during home interviews using the Dutch version of the CES-D scale (Beekman et al., 1997; Radloff, 1977). The questionnaire, designed for research in the general population, consists of 20 questions referring to the presence and severity of depressive symptoms during the past week, such as ‘I felt depressed’ or ‘I could not get going’. Answer options range from zero (‘rarely or none of the time’) to three (‘most or all of the time’), resulting in a sum score between 0 and 60, with higher scores indicating more depressive symptoms. A validated cut-off score of 16 points or higher on the CES-D scale is considered indicative of clinically relevant depressive symptoms (Beekman et al., 1997). Depressive symptoms during a period in the COVID-19 pandemic (April 2020–July 2020) were assessed by survey using a validated shortened version of the CES-D scale, consisting of 10 items, with a sum score ranging from 0 to 30 and a commonly used cut-off score of 10 points or higher (Andresen et al., 1994; Mooldijk et al., 2022; van den Besselaar et al., 2021).

##### Longitudinal data

Depression-related phenotypes were identified using three information sources, as previously described in detail (Luijendijk et al., 2008). First, the CES-D scale was assessed during the home interview at baseline and at each follow-up measurement. Second, participants screening positive for depressive symptoms on the CES-D scale were invited for a clinical semi-structured interview by trained personnel: The Dutch version of the Schedules for Clinical Assessment in Neuropsychiatry (SCAN). Based on the interview, depressive disorders were diagnosed and classified according to the Statistical Manual of Mental Disorders, Fourth revised edition (DSM-IV-TR). Third, monitoring of medical records for the occurrence of depression-related phenotypes took place from baseline onward. To assess the incidence of depression-related phenotypes during the follow-up period, trained research assistants reviewed the medical records of general practitioners, which include not only general practitioners’ notes but also letters from hospitals, specialists, and psychologists/psychiatrists. Each medical record was reviewed by two trained research assistants, and depressive episodes were categorized according to a predefined protocol. Disagreeing categorizations were discussed in consensus meetings. If no depression-related phenotype was identified, participants were censored if they moved, at death, at January 1, 2012 (RS-I and RS-II), or at January 1, 2019 (RS-III).

From these sources, three depression-related phenotypes were derived, ordered by increasing severity: (1) clinically relevant depressive symptoms (based on SCAN or clinician-reported symptoms); (2) depressive syndromes (DSM-IV-TR defined cases of dysthymic disorder, mood disorder due to medical condition, and mood disorder not otherwise specified); and (3) major depressive disorder (DSM-IV-TR defined, single or recurrent) (Luijendijk et al., 2008).

We defined ‘any depression-related phenotype’ as the occurrence of at least one phenotype during follow-up. For assessment per phenotype, we categorized each participant based on the most severe phenotype during follow-up.

##### Covariates

Self-report data were available on sex and age.

### Statistical analysis

The study sample was characterized using descriptive statistics. Pearson’s correlations between GRS and PRS were assessed for cases and non-cases based on any depression-related phenotype, and separately for each phenotype.

Generalized linear models (GLM) were used to estimate the cross-sectional association of the continuous GRS and PRS for depression with depressive symptoms (CES-D score). To assess whether genetic risk is longitudinally associated with the occurrence of depression-related phenotypes at middle and older age, binomial GLMs were used to estimate the association of the continuous GRS and PRS with any depression-related phenotype, and multinomial models to estimate the associations with the type of phenotype (i.e. clinically relevant depressive symptoms, depressive syndrome, and major depressive disorder). To establish relative risks for individuals surpassing specific percentiles of the risk score distribution, participants were grouped by their risk score percentile (i.e. 5^th^, 10^th^, 20^th^, 25^th^, 75^th^, 80^th^, 90^th^, or 95^th^ percentiles). Each group was compared to the middle 50% of the relevant risk score distribution. Lastly, linear models were used to assess the association of GRS and PRS with depressive symptoms during the COVID-19 pandemic. We also explored the models for any depression-related phenotype in different age strata (45–54 years, 55–64 years, 65–74 years, and 75 years and older). All models were adjusted for sex. As the study population represented a genetically homogeneous European ancestry group, population structure did not warrant adjustment, as previously described (Ikram et al., 2024). All analyses were performed per subcohort and then meta-analyzed (Meta). Fixed-effects models were used as the three subcohorts originate from the same district in Rotterdam and represent subpopulations of the population-based Rotterdam Study. Data were handled and analyzed using R version 4.0.2 with ‘rmeta’, ‘nnet’, and ‘ggplot2’ packages (The R Foundation for Statistical Computing, Vienna, Austria).

## Results

Table 1 (and Supplementary Table S1) presents study sample characteristics. The mean follow-up time for subcohort RS-I (N = 4,312) was 12.6 years (standard deviation [SD]: 5.3 years), RS-II (N = 2,063) 10.1 years (SD: 2.4 years), and RS-III (N = 2,823) 10.3 years (SD: 2.2 years).

**Table 1.** Characteristics of the study sample

	Cross-sectional data sample (N = 7316)			Longitudinal data sample (N = 9198)		
	RS-I (N = 2700)	RS-II (N = 1779)	RS-III (N = 2837)	RS-I (N = 4312)	RS-II (N = 2063)	RS-III (N = 2823)
Female	1604 (59.4%)	985 (55.4%)	1591 (56.1%)	2495 (57.9%)	1119 (54.2%)	1585 (56.1%)
Age of measurement*	75.8 ± 6.3	68.2 ± 7.4	57.3 ± 6.9	67.3 ± 7.8	64.7 ± 7.9	57.3 ± 6.9
GRS for depression	−5.97 ± 0.28 e–04	−5.80 ± 0.28 e–04	−5.80 ± 0.28 e–04	−5.96 ± 0.29 e–04	−5.80 ± 0.28 e–04	−5.80 ± 0.28 e–04
PRS for depression	0.16 ± 0.14	0.16 ± 0.14	0.16 ± 0.14	0.16 ± 0.14	0.16 ± 0.14	0.16 ± 0.14
CES-D score *median (IQR)*	5.0 (1.0–11.0)	3.0 (1.0–8.0)	3.0 (1.0–7.0)			
CES-D score > 16	384 (14.2%)	184 (10.3%)	270 (9.5%)			
Any depression-related phenotype				1200 (27.8%)	531 (25.7%)	625 (22.1%)
Depressive symptoms				1019 (23.6%)	410 (19.9%)	477 (16.9%)
Depressive syndrome				173 (4.0%)	93 (4.5%)	136 (4.8%)
Major depressive disorder				197 (4.6%)	131 (6.3%)	134 (4.7%)
Age at first depression-related phenotype *during FU*				75.3 ± 8.2	68.8 ± 9.1	59.2 ± 7.1
Number of depression-related phenotypes *during FU* 1 phenotype > 1 phenotype				808 (18.7%) 392 (9.1%)	349 (16.9%) 182 (8.8%)	411 (14.6%) 214 (7.6%)

*Note:* Data are presented as N (%) or mean ± SD unless otherwise indicated. *Age of measurement refers to the date of interview for cross-sectionally measured data, and the start date of follow-up (FU) for longitudinal data. RS = Rotterdam Study, referring to subcohort, SD = standard deviation, GRS = genome-wide risk score, PRS = polygenic risk score, CES-D = Center for Epidemiological Studies – Depression scale, IQR = interquartile range.

To compute the GRS based on the best-fitting model, we opted for a cutoff of p < 0.05 as the preferred threshold across three RS subcohorts. This choice was made because it yielded the highest variance explained in RS-I (R^2^ = 8.1x10^−7^) and RS-III (R^2^ = 1.4x10^−8^). However, for RS-II, the highest variance explained was achieved with a cutoff of p < 0.5 (R^2^ = 1.0x10^−3^) (Supplementary Figure S1). The unstandardized GRS and PRS showed similar distributions across subcohorts (Table 1). Correlations between the GRS and PRS were *r ≤* 0.20 (Supplementary Figure S2).

### Genetic risk scores and associations with cross-sectionally measured depressive symptoms

**Figure 1.**
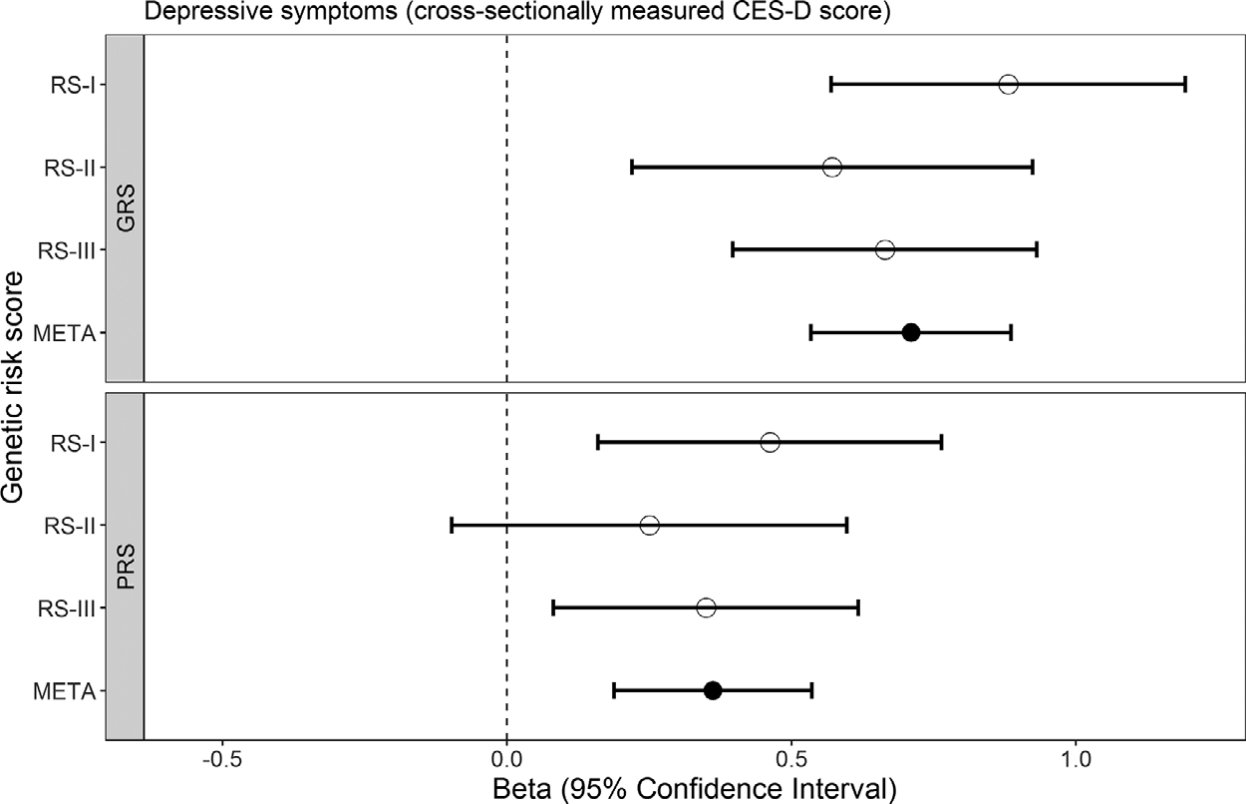
Associations of the GRS for depression (top panel) and the PRS for depression (bottom panel) with cross-sectionally measured depressive symptoms. CES-D refers to the Center for Epidemiological Studies – Depression scale, RS to Rotterdam Study (subcohort), GRS to genome-wide risk score, PRS to polygenic risk score, and META to meta-analysis.

**Figure 2.**
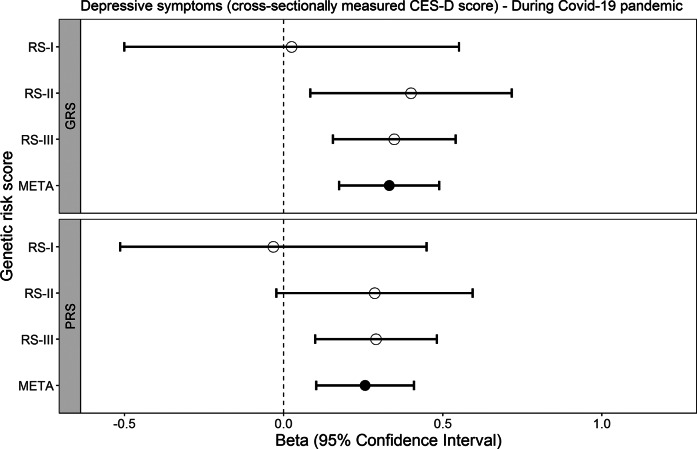
Associations of the GRS for depression (top panel) and the PRS for depression (bottom panel) with cross-sectionally measured depressive symptoms during the COVID-19 pandemic. CES-D refers to the Center for Epidemiological Studies – Depression scale, RS to Rotterdam Study (subcohort), GRS to genome-wide risk score, PRS to polygenic risk score, and META to meta-analysis.

One standard deviation higher GRS for depression was associated with a 0.71 points higher cross-sectionally measured depressive symptoms score on the CES-D (estimated difference β_GRS_ = 0.71, 95% confidence interval [0.53–0.89]), and one standard deviation higher PRS for depression with a 0.36 points higher score (β_PRS_ = 0.36 [0.19–0.54], Figure 1, Supplementary Table S2). Both GRS and PRS also showed a positive association with a depressive symptoms score above the validated cut-off, indicating clinically relevant depressive symptoms (Odds Ratio (OR)_GRS_ = 1.29 [1.20–1.39]; OR_PRS_ = 1.13 [1.05–1.22], Supplementary Table S2). For depressive symptoms measured during the COVID-19 pandemic using the shortened version (scale 0–30), higher GRS and PRS were also associated with higher CES-D scores (β_GRS_ = 0.33 [0.17–0.49], β_PRS_ = 0.26 [0.10–0.41], Figure 2, Supplementary Table S3).

### Genetic risk scores and associations with longitudinally measured phenotypes of depression

**Figure 3.**
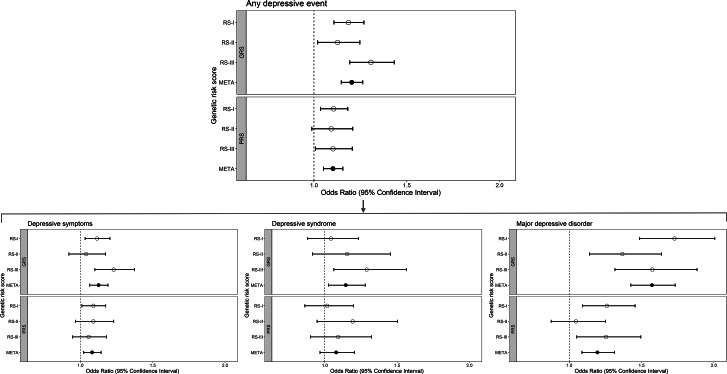
Associations of the GRS for depression (top panels) and the PRS for depression (bottom panels) with longitudinally measured depression-related phenotypes: any depression-related phenotype, and then categorized into worst phenotype, that is depressive symptoms, depressive syndrome, or major depressive disorder. RS refers to Rotterdam Study (subcohort), GRS to genome-wide risk score, PRS to polygenic risk score, and META to meta-analysis.

The standardized GRS and PRS for depression were significantly associated with any depression-related phenotype during follow-up (OR_GRS_ = 1.20 [1.15–1.26], OR_PRS_ = 1.10 [1.05–1.16], Figure 3, Supplementary Table S4). When assessing per phenotype (clinically relevant depressive symptoms, depressive syndromes, and major depressive disorder), we observed the highest effect size for episodes of major depressive disorder (OR_GRS_ = 1.57 [1.43–1.73], OR_PRS_ = 1.19 [1.09–1.31]), while the effect sizes for clinically relevant depressive symptoms (OR_GRS_ = 1.13 [1.06–1.19], OR_PRS_ = 1.08 [1.02–1.14]) and depressive syndrome (OR_GRS_ = 1.14 [1.02–1.28], OR_PRS_ = 1.08 [0.97–1.20]) were more similar (Figure 3, Supplementary Table S4). When these associations for any depression-related phenotype were studied by 10-year age groups, effect estimates for both the GRS and PRS were consistent in direction and comparable in size across participants aged 45–74 years (Supplementary Table S5). In the oldest group (75+ years), estimates from meta-analyses were smaller and non-significant, but estimates per cohort differed in direction and were based on a limited number of cases.

### Relative risks of any depression-related phenotype in the tails of the genetic risk score distributions

Participants in the lower and upper tails of the GRS distribution (25%, 20%, 10%, and 5%) showed significantly different risks of any depression-related phenotype compared to the middle 50% of the distribution (e.g. OR_GRS,90%_ = 1.33 [1.14–1.55], OR_GRS,95%_ = 1.46 [1.18–1.79]). Again, effect sizes were largest for episodes of major depressive disorder (e.g. OR_GRS,90%_ = 1.99 [1.53–2.57], OR_GRS,95%_ = 2.22 [1.58–3.06]). All lower and upper tails (25% and smaller) of the GRS distribution were significantly associated with each depression-related phenotype, except that all upper tails of the GRS distribution did not show a significantly higher risk for depressive syndrome (Supplementary Figure S3, Supplementary Table S6).

For the PRS distribution, similar trends were observed, although effect sizes were smaller and not consistently statistically significant across all lower and upper tails (e.g. for any depression-related phenotype: OR_PRS,90%_ = 1.26 [1.08–1.48], OR_PRS,95%_ = 1.16 [0.94–1.44]). Also for PRS, effect sizes were largest for major depressive disorder, but not all statistically significant (e.g. OR_PRS,90%_ = 1.51 [1.13–1.99], OR_PRS,95%_ = 1.45 [0.98–2.09], Supplementary Figure S3, Supplementary Table S6).

## Discussion

Our findings indicated that a GRS for depression was associated with depression-related phenotypes in this population-based cohort of middle-aged and older adults, including cross-sectional, longitudinal, and COVID-19-related outcomes. Effect sizes varied slightly across these different outcomes. The PRS for depression showed similar patterns, with generally more modest associations.

Our results indicate that higher GRS and PRS for depression are more strongly associated with major depressive disorder, compared to clinically depressive symptoms or syndrome. This likely reflects the phenotype definitions used in the GWAS study (Howard et al., 2019), ranging from depressive symptom scales to clinical diagnoses. Consequently, cases of major depressive disorder were probably captured across all cohorts, while less severe cases were only included in certain cohorts, potentially tailoring the risk scores to predict risk of more severe cases of depression. Genetic assessment might benefit from GWAS that differentiate between phenotypes (e.g. based on severity) (Mitchell et al., 2021), although this remains challenging due to the heterogeneous presentation of depression-related phenotypes (Olbert et al., 2014). Also, due to greater heterogeneity and stronger influence of environmental factors, depressive symptoms might be less heritable than major depressive disorder (Fried et al., 2014; Jang et al., 2004). This may also explain the smaller effect sizes for continuous CES-D scores. Although statistically significant, a difference of 0.71 points on the CES-D scale per SD in GRS/PRS might be of uncertain clinical relevance. Overall, these findings might suggest greater potential for genetic risk scores for identifying individuals at risk of clinically relevant depression, rather than capturing subtle variation in symptom levels.

Our results suggest that genetic risk scores (GRS and PRS), based on a large GWAS study (Howard et al., 2019), show modest associations with depression-related phenotypes in middle-aged and older adults. This aligns with previous studies reporting modest effect sizes and limited explained variance (Halldorsdottir et al., 2019; Howard et al., 2019; Kosciuszko et al., 2023; Musliner et al., 2019). More specifically, a comparison with the validation cohorts in Howard et al. is possible: the odds ratio for MDD in the top versus bottom 10% of our GRS was 4.2, which is similar to the estimates reported in their study (ORs ranging from 2.0 to 3.5 across cohorts and p-value thresholds). Although the original GWAS included studies with broad age ranges across adulthood, older adults may have been underrepresented. Nevertheless, our findings suggest the derived genetic risk scores associate with depression-related phenotypes in a sample of middle-aged and older adults. Within our cohort, effect sizes for the GRS and PRS were consistent across age groups between 45 and 74 years. In the oldest group (75+), associations appeared weaker, but low event numbers and heterogeneity between cohorts limit interpretation. Although studies allowing for more direct comparisons across adulthood are needed, our and prior findings do not indicate that the role of genetic risk meaningfully changes with age.

Genetic risk scores were associated with cross-sectional and longitudinal ascertained phenotypes, even as depressive symptoms during the COVID-19 pandemic. This supports the potential applicability of genetic risk scores across various depression characteristics and settings. Associations present during the COVID-19 pandemic align with studies linking genetic risk scores to psychiatric symptoms following specific triggers or events (Kwong et al., 2021b; Waszczuk et al., 2020). Direct comparison with pre-pandemic results is complicated by population (participants with available data during the pandemic were older on average) and measurement differences (full vs. shortened version of the CES-D; interview vs. survey data). Among older Dutch adults, self-reported symptoms tend to be higher than data from interviews (Geerlings et al., 1999). Therefore, we could not determine whether the role of genetic predisposition is potentially stronger or weaker in the context of an external stressor such as the pandemic. Nonetheless, our findings suggest that cross-sectional depression measures may capture similar genetic associations as lifetime prevalence, which may be relevant for genetic research settings.

Regarding the two genetic risk scores we used, a notable trend emerges when comparing PRS and GRS: the effect sizes for genome-wide scores (GRS) tend to surpass those of restricted scores (PRS), although most differences were not statistically significant. The broader range of genetic variants included in the GRS likely contributed to its slightly better performance. For the GRS, we selected the p-value threshold that maximized explained variance in our study, although this variance remained very low across all thresholds and was not fully consistent across cohorts. This underscores the limited predictive power of current approaches for depression and highlights that optimal thresholds may be population specific. In contrast, PRS includes only a select set of the most significantly associated variants, which may facilitate biological interpretation and clinical translation. Particularly, biological annotation and interpretation of a restricted PRS could be used to explore the underlying disease mechanisms (Schreurs et al., 2024; Stikker et al., 2024), although we acknowledge that this approach may oversimplify the biological complexity of depression by ignoring the thousands of more subtle associated SNPs. Future studies should consider advanced polygenic scoring methods such as SBayesRC or LDpred-funct, which integrate functional annotations and genome-wide LD modeling, thereby offering enhanced predictive accuracy and the potential for deeper biological insights.

In psychiatric phenotypes, genetic risk factors might ultimately contribute to early risk detection, accurate diagnosis, and personalized treatment strategies (Murray et al., 2021). However, as genetic factors account for only a small part of the overall risk of psychiatric disorders and the variance explained by genetic risk scores is generally low, interpretation and communication of these results remain challenging. A more comprehensive and strongly associated risk score may be needed before application in clinical practice. Additionally, given the lack of a clear prevention strategy for psychiatric disorders, it is important to consider the potential applications and consequences of knowing an individual’s genetic risk. Understanding these factors is crucial before incorporating and communicating these scores in clinical practice (Murray et al., 2021; Wray et al., 2021).

Strengths of this study include the population-based character of our sample, the inclusion of multiple and extensive measurements of depression, and the comparison of two common types of genetic risk scores. However, several limitations should be mentioned. First, the sample size was relatively small in some analyses, e.g. the analyses by age group or the RS-I subcohort in the COVID-19 data analyses. Second, the risk scores that we used were based on a GWAS including various definitions of depression rather than specific depressive (sub)phenotypes. Third, although we used one of the most extensive GWAS studies available so far, the explained variance of the PRS is still small (ranging from 1.5 to 3.2%) (Howard et al., 2019). Probably, future GWAS studies will enable genetic risk scores with higher explained variance, as shown by the available preprint of McIntosh et al. (explained variance of 5.7%) (McIntosh & Lewis, 2024). Fourth, the risk scores we constructed were based on GWAS in European populations. Therefore, our findings are only applicable to individuals of European ancestry. Fifth, we were unable to assess the effects of antidepressant use; future sensitivity analyses should address this, while considering potential collider bias. Finally, our study focused on genetic risk only, while in real-life settings, it might be important to consider genetic risk in interaction with other individual and environmental risk factors.

## Conclusions

In a population of middle-aged and older adults, both the GRS and, to a smaller extent, the PRS for depression showed modest associations with a range of depression-related phenotypes. Effect sizes for both GRS and PRS seem to increase with the severity of depression. Based on cross-sectional and longitudinal measured data, the GRS and PRS are associated with both prevalent and incident depression. These findings support the potential relevance of genetic risk scores in understanding vulnerability to depression. However, their explained variance remains limited, emphasizing the need for further improvement before their clinical utility can be meaningfully assessed.

## Supporting information

Hofman et al. supplementary material Hofman et al. supplementary material

## Data Availability

Data can be obtained upon request. Requests should be directed toward the management team of the Rotterdam Study (secretariat.epi@erasmusmc.nl), which has a protocol for approving data requests. Because of restrictions based on privacy regulations and informed consent of the participants, data cannot be made freely available in a public repository.

## Supplementary material

The supplementary material for this article can be found at http://doi.org/10.1017/S0033291725101943.
