# Supplementary material for: Genetic risk scores of psychiatric phenotypes are associated with depression risk in a prospective Dutch population-based cohort
Source: Psychol Med. 2025 Sep 29;55:e288. doi: 10.1017/S0033291725101943 (PMC12527510; doi:10.1017/S0033291725101943)
Supplement: Hofman et al. supplementary material [file S0033291725101943sup001.docx]

**Supplemental Material**

**Table S1.** Characteristics of the study sample that participated during the Covid-19 pandemic

|  | Covid-19 data sample (N=2976) | | |
| --- | --- | --- | --- |
|  | RS-I (N=395) | RS-II (N=767) | RS-III (N=1814) |
| Female | 231 (58.5%) | 403 (52.5%) | 1056 (58.2%) |
| Age of measurement | 86.9 ± 3.7 | 79.5 ± 3.5 | 68.8 ± 5.3 |
| GRS for depression | -5.98 ± 0.27 e-04 | -5.81 ± 0.28 e-04 | -5.81 ± 0.27 e-04 |
| PRS for depression | 0.17 ± 0.14 | 0.15 ± 0.14 | 0.16 ± 0.14 |
| CES-D score* *median (IQR)* | 5.0 (2.0 - 9.0) | 4.0 (2.0 - 8.0) | 4.0 (1.0 - 7.0) |
| CES-D score > 16 | 86 (21.8%) | 129 (16.8%) | 243 (13.4%) |

Note: Data are presented as N(%) or mean(SD) unless otherwise indicated. *A shortened version of the CES-D questionnaire was used in this sample. RS = Rotterdam Study, referring to subcohort, SD = standard deviation, GRS = genome-wide risk score, PRS = restricted polygenic risk score, CES-D = Center for Epidemiological Studies - Depression scale, IQR = interquartile range.

**Table S2.** Associations of the GRS and PRS for depression with depressive symptoms, based on cross-sectionally measured data.

|  | CES-D Continuous score | | | | CES-D > 16 | | | |
| --- | --- | --- | --- | --- | --- | --- | --- | --- |
|  | RS-I (N=2700) | RS-II (N=1779) | RS-III (N=2837) | Meta-analysis | RS-I (N=2700) | RS-II (N=1779) | RS-III (N=2837) | Meta-analysis |
|  | β (95%CI) | β (95%CI) | β (95%CI) | β (95%CI) | OR (95%CI) | OR (95%CI) | OR (95%CI) | OR (95%CI) |
| GRS for depression | 0.88 (0.57;1.19) | 0.57 (0.22;0.92) | 0.66 (0.40;0.93) | 0.71 (0.53;0.89) | 1.26 (1.13;1.41) | 1.28 (1.09;1.49) | 1.35 (1.18;1.53) | 1.29 (1.20;1.39) |
| PRS for depression | 0.46 (0.16;0.76) | 0.25 (-0.10;0.60) | 0.35 (0.08;0.62) | 0.36 (0.19;0.54) | 1.15 (1.03;1.28) | 1.12 (0.96;1.30) | 1.12 (0.98;1.27) | 1.13 (1.05;1.22) |

Note: CES-D = Center for Epidemiological Studies - Depression scale, RS = Rotterdam Study, referring to subcohort, OR = Odds Ratio, CI = Confidence Interval, GRS = genome-wide risk score, PRS = restricted polygenic risk score.

**Table S3.** Associations of the GRS for depression with depressive symptoms, based on cross-sectionally measured data during Covid-19 pandemic.

|  | CES-D Continuous score | | | | CES-D > 16 | | | |
| --- | --- | --- | --- | --- | --- | --- | --- | --- |
|  | RS-I (N=386) | RS-II (N=767) | RS-III (N=1802) | Meta-analysis | RS-I (N=386) | RS-II (N=767) | RS-III (N=1802) | Meta-analysis |
|  | β (95%CI) | β (95%CI) | β (95%CI) | β (95%CI) | OR (95%CI) | OR (95%CI) | OR (95%CI) | OR (95%CI) |
| GRS for depression | 0.03 (-0.50;0.55) | 0.40 (0.08;0.72) | 0.35 (0.15;0.54) | 0.33 (0.17;0.49) | 1.10 (0.86;1.42) | 1.23 (1.01;1.50) | 1.11 (0.97;1.27) | 1.14 (1.03;1.26) |
| PRS for depression | -0.03 (-0.51;0.45) | 0.29 (-0.02;0.59) | 0.29 (0.10;0.48) | 0.26 (0.10;0.41) | 0.86 (0.68;1.09) | 1.16 (0.96;1.40) | 1.17 (1.02;1.34) | 1.11 (1.00;1.22) |

Note: CES-D = Center for Epidemiological Studies - Depression scale, RS = Rotterdam Study, referring to subcohort, OR = Odds Ratio, CI = Confidence Interval, GRS = genome-wide risk score, PRS = restricted polygenic risk score.

**Table S4.** Associations of the GRS and PRS for depression with any event of depression, and then categorized into worst event, i.e., depressive symptoms, depressive syndrome or major depressive disorder.

|  | RS-I (N=4312) | RS-II (N=2063) | RS-III (N=2823) | Meta-analysis |
| --- | --- | --- | --- | --- |
|  | OR (95%CI) | OR (95%CI) | OR (95%CI) | OR (95%CI) |
| Any depressive event | | | | |
| GRS for depression | 1.19 (1.11; 1.27) | 1.13 (1.02; 1.25) | 1.31 (1.19; 1.43) | 1.20 (1.15; 1.26) |
| PRS for depression | 1.11 (1.03; 1.18) | 1.09 (0.99; 1.21) | 1.10 (1.01; 1.21) | 1.10 (1.05; 1.16) |
| Depressive symptoms |  |  |  |  |
| GRS for depression | 1.12 (1.03; 1.20) | 1.04 (0.92; 1.17) | 1.23 (1.10; 1.37) | 1.13 (1.06; 1.19) |
| PRS for depression | 1.09 (1.01; 1.17) | 1.09 (0.97; 1.23) | 1.06 (0.95; 1.18) | 1.08 (1.02; 1.14) |
| Depressive syndrome |  |  |  |  |
| GRS for depression | 1.04 (0.88; 1.23) | 1.15 (0.92; 1.45) | 1.29 (1.06; 1.56) | 1.14 (1.02; 1.28) |
| PRS for depression | 1.02 (0.86; 1.20) | 1.19 (0.95; 1.50) | 1.09 (0.90; 1.32) | 1.08 (0.97; 1.20) |
| Major depressive disorder |  |  |  |  |
| GRS for depression | 1.73 (1.49; 2.01) | 1.37 (1.14; 1.64) | 1.57 (1.32; 1.88) | 1.57 (1.43; 1.73) |
| PRS for depression | 1.26 (1.09; 1.45) | 1.05 (0.87; 1.25) | 1.25 (1.05; 1.49) | 1.19 (1.09; 1.31) |

Note: RS = Rotterdam Study, referring to subcohort, OR = Odds Ratio, CI = Confidence Interval, GRS = genome-wide risk score, PRS = restricted polygenic risk score.

**Table S5.** Associations of the GRS for depression with any event of depression, stratified by age group.

|  | RS-I | RS-II | RS-III | Meta-analysis |
| --- | --- | --- | --- | --- |
|  | OR (95%CI) | OR (95%CI) | OR (95%CI) | OR (95%CI) |
| Total (n/N) | **1200 / 4312** | **531 / 2063** | **625 / 2823** | **2356 / 9198** |
| GRS for depression | 1.19 (1.11; 1.27) | 1.13 (1.02; 1.25) | 1.31 (1.19; 1.43) | 1.20 (1.15; 1.26) |
| PRS for depression | 1.11 (1.03; 1.18) | 1.09 (0.99; 1.21) | 1.10 (1.01; 1.21) | 1.10 (1.05; 1.16) |
| 45 – 54 years (n/N) |  |  | **266 / 1077** |  |
| GRS for depression |  |  | 1.28 (1.10; 1.48) |  |
| PRS for depression |  |  | 1.03 (0.90; 1.19) |  |
| 55 – 64 years (n/N) | **511 / 1893** | **332 / 1367** | **311 / 1505** | **1154 / 4765** |
| GRS for depression | 1.18 (1.06; 1.31) | 1.18 (1.04; 1.34) | 1.33 (1.17; 1.51) | 1.22 (1.14; 1.31) |
| PRS for depression | 1.10 (0.99; 1.21) | 1.08 (0.95; 1.22) | 1.13 (1.00; 1.29) | 1.10 (1.03; 1.18) |
| 65 – 74 years (n/N) | **502 / 1672** | **107 / 404** | **26 / 149** | **635 / 2225** |
| GRS for depression | 1.24 (1.12; 1.38) | 1.29 (1.03; 1.63) | 1.37 (0.91; 2.11) | 1.25 (1.14; 1.38) |
| PRS for depression | 1.15 (1.04; 1.28) | 1.22 (0.98; 1.52) | 1.12 (0.70; 1.80) | 1.16 (1.06; 1.28) |
| 75+ years (n/N) | **187 / 747** | **92 / 292** | **22 / 92** | **301 / 1131** |
| GRS for depression | 1.10 (0.93; 1.30) | 0.81 (0.63; 1.04) | 1.50 (0.89; 2.66) | 1.03 (0.90; 1.18) |
| PRS for depression | 1.02 (0.86; 1.20) | 0.99 (0.75; 1.30) | 1.51 (0.92; 2.56) | 1.04 (0.91; 1.19) |

Note: RS = Rotterdam Study, referring to subcohort, OR = Odds Ratio, CI = Confidence Interval, GRS = genome-wide risk score, PRS = restricted polygenic risk score.

**Table S6.** Associations of the GRS and PRS for depression, based on percentiles of the population distribution, with any event of depression, and then categorized into worst event, i.e., depressive symptoms, depressive syndrome or major depressive disorder.

|  | Any event | Depressive symptoms | Depressive syndrome | Major depressive disorder |
| --- | --- | --- | --- | --- |
|  | OR (95%CI) | OR (95%CI) | OR (95%CI) | OR (95%CI) |
| GRS | | | | |
| Lowest 5% vs middle 50% | 0.61 (0.47; 0.78) | 0.67 (0.51; 0.88) | 0.41 (0.19; 0.75) | 0.22 (0.08; 0.47) |
| Lowest 10% vs middle 50% | 0.70 (0.59; 0.84) | 0.73 (0.60; 0.88) | 0.66 (0.44; 0.96) | 0.48 (0.30; 0.73) |
| Lowest 20% vs middle 50% | 0.79 (0.70; 0.90) | 0.82 (0.71; 0.94) | 0.75 (0.56; 0.99) | 0.54 (0.39; 0.73) |
| Lowest 25% vs middle 50% | 0.76 (0.67; 0.86) | 0.79 (0.69; 0.90) | 0.67 (0.51; 0.87) | 0.58 (0.44; 0.76) |
| Highest 25% vs middle 50% | 1.20 (1.07; 1.34) | 1.17 (1.04; 1.32) | 1.02 (0.80; 1.29) | 1.54 (1.25; 1.90) |
| Highest 20% vs middle 50% | 1.22 (1.08; 1.38) | 1.16 (1.01; 1.32) | 1.03 (0.80; 1.33) | 1.67 (1.34; 2.07) |
| Highest 10% vs middle 50% | 1.33 (1.14; 1.55) | 1.22 (1.03; 1.44) | 1.10 (0.79; 1.51) | 1.99 (1.53; 2.57) |
| Highest 5% vs middle 50% | 1.46 (1.18; 1.79) | 1.41 (1.13; 1.76) | 1.10 (0.69; 1.65) | 2.22 (1.58; 3.06) |
| Highest 10% vs lowest 10%* | 1.90 (1.53; 2.36) | 1.67 (1.32; 2.11) | 1.68 (1.05; 2.71) | 4.16 (2.63; 6.87) |
| PRS |  |  |  |  |
| Lowest 5% vs middle 50% | 0.97 (0.77; 1.21) | 1.04 (0.82; 1.32) | 0.88 (0.52; 1.40) | 0.73 (0.42; 1.16) |
| Lowest 10% vs middle 50% | 0.90 (0.76; 1.06) | 0.97 (0.81; 1.16) | 0.88 (0.60; 1.24) | 0.83 (0.58; 1.17) |
| Lowest 20% vs middle 50% | 0.87 (0.76; 0.99) | 0.92 (0.80; 1.06) | 0.87 (0.66; 1.15) | 0.79 (0.60; 1.03) |
| Lowest 25% vs middle 50% | 0.88 (0.79; 1.00) | 0.93 (0.82; 1.06) | 0.94 (0.73; 1.20) | 0.79 (0.61; 1.01) |
| Highest 25% vs middle 50% | 1.18 (1.05; 1.32) | 1.19 (1.05; 1.34) | 1.04 (0.81; 1.32) | 1.25 (1.01; 1.55) |
| Highest 20% vs middle 50% | 1.18 (1.04; 1.33) | 1.18 (1.03; 1.34) | 1.05 (0.80; 1.35) | 1.23 (0.97; 1.56) |
| Highest 10% vs middle 50% | 1.26 (1.08; 1.48) | 1.24 (1.05; 1.47) | 0.99 (0.69; 1.39) | 1.51 (1.13; 1.99) |
| Highest 5% vs middle 50% | 1.16 (0.94; 1.44) | 1.11 (0.88; 1.40) | 1.13 (0.71; 1.72) | 1.45 (0.98; 2.09) |
| Highest 10% vs lowest 10%* | 1.41 (1.14; 1.74) | 1.28 (1.02; 1.60) | 1.14 (0.72; 1.81) | 1.82 (1.22; 2.76) |

Note: OR = Odds Ratio, CI = Confidence Interval, GRS = genome-wide risk score, PRS = restricted polygenic risk score. *Results for highest 10% versus lowest 10% were added to allow comparison with results of Howard et al. (2019). These results were not shown in corresponding plots (Figure S3).

**
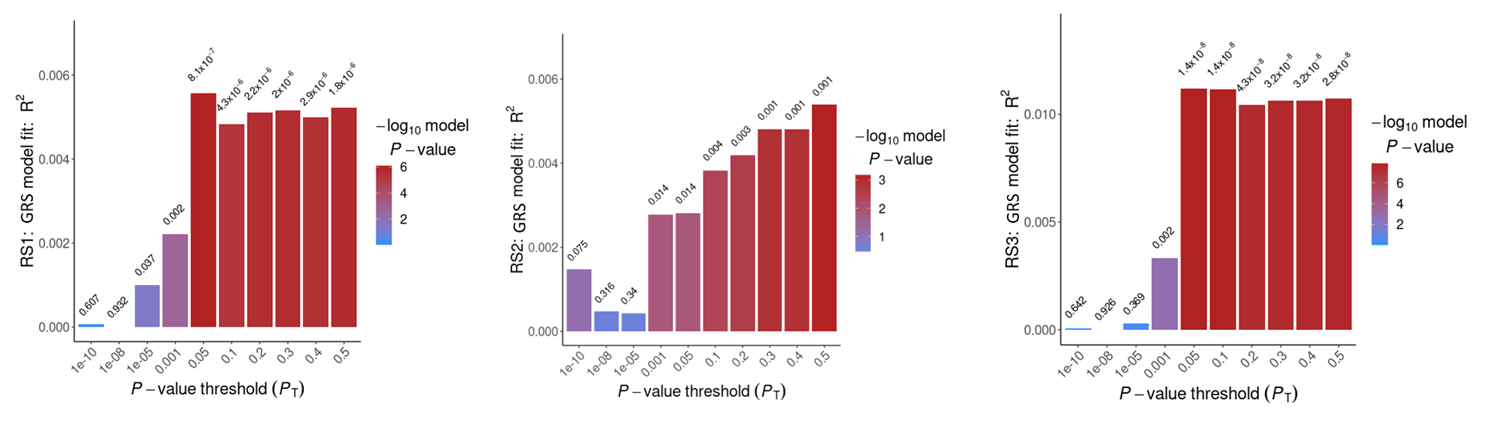
Figure S1.** Variance explained for GRS based on different p-value thresholds. RS refers to Rotterdam Study subcohort, GRS to genome-wide risk score.

**
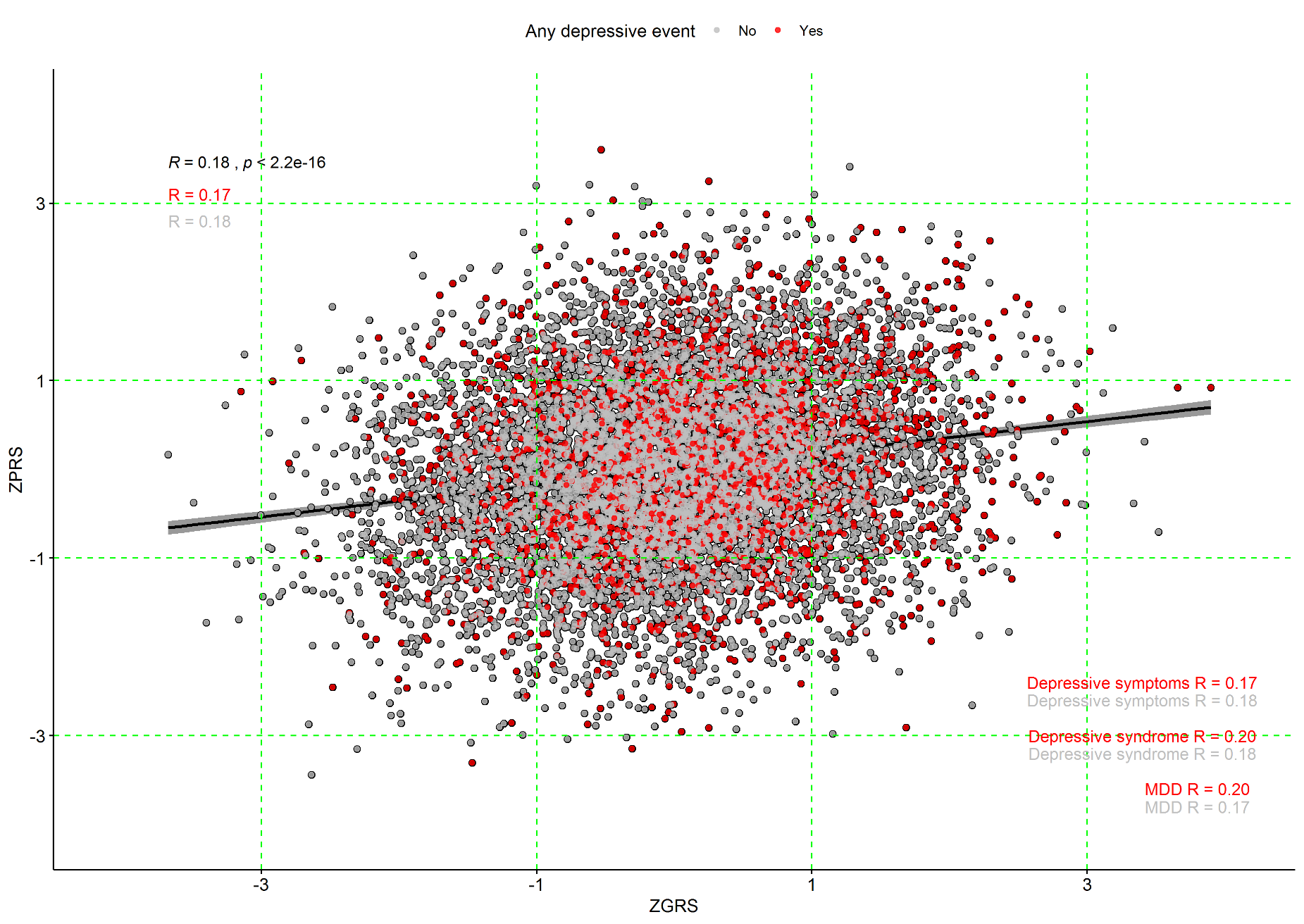
**

**Figure S2.** Correlations between the GRS for depression and the PRS for depression, for cases and non-cases. The figure represents the correlation plot for any event of depression, in the right bottom corner correlation coefficients for each type of event are presented. GRS refers to genome-wide risk score, PRS to restricted polygenic risk score, MDD to major depressive disorder.

**
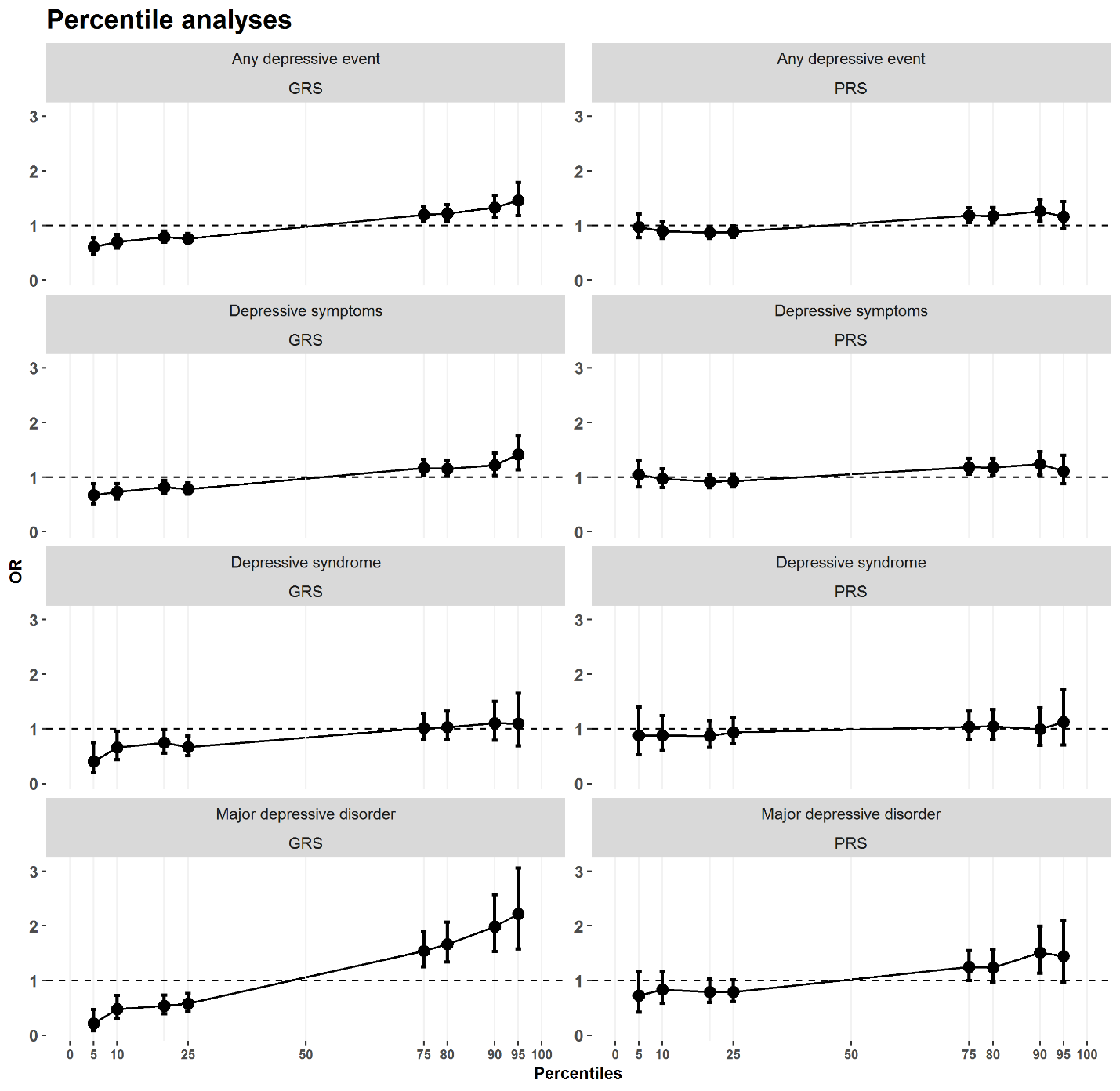
Figure S3.** The association between percentiles of genetic risk scores and any depressive event, based on longitudinal data (percentiles compared to the middle 50% as reference group). GRS refers to genome-wide risk score, PRS to restricted polygenic risk score, OR to odds ratio.
